# How breastfeeding behavior develops in women with gestational diabetes mellitus: A qualitative study based on health belief model in China

**DOI:** 10.3389/fendo.2022.955484

**Published:** 2022-10-03

**Authors:** Pan Qian, Lixia Duan, Rujiao Lin, Xiwang Du, Dan Wang, Chenxi Liu, Tieying Zeng

**Affiliations:** ^1^ Nursing Department in Tongji Hospital, Tongji Medical School, Huazhong University of Science and Technology, Wuhan, China; ^2^ School of Medical and Health Management, Tongji Medical School, Huazhong University of Science and Technology, Wuhan, China

**Keywords:** gestational diabetes mellitus, breastfeeding, health belief model, qualitative design, China

## Abstract

**Background:**

Gestational diabetes mellitus (GDM) is a condition in which women develop hyperglycemia during pregnancy, and is associated with long-term health burden on both mother and their offspring, such as future type 2 diabetes mellitus (T2DM). Although breastfeeding was expected to mitigate metabolic sequelae for both mothers and their newborns, the prevalence of breastfeeding in GDM mothers are sub-optimal worldwide.

**Objective:**

To explore the experience of disease among mothers with GDM and how they develop feeding behaviors.

**Methods:**

This study was conducted in three branches of an integrated tertiary hospital in the central area of China. Mothers who were diagnosed with GDM, had no other complications, and gave birth before no more than 6 months were approached based on a purposive sampling. GDM mothers’ experience of the disease and breastfeeding were collected *via* in-depth interviews. A theory-driven thematic analysis based on Health Belief Model (HBM) was applied for data analysis. Inductive reasoning was used to identify emerging themes which were not included in HBM.

**Results:**

16 GDM mothers were included in the current study, with nine using breastfeeding, six mixed feeding and one artificial feeding, respectively. Nine themes were identified, including: 1) GDM diagnosis and severity; 2) information searching and GDM knowledge;3) GDM management; 4) perceived susceptibility of future diabetes;5) perceived severity of future diabetes;6) perceived benefits of breastfeeding;7) perceived barriers of breastfeeding;8) decision making process of feeding and social support. Generally, mothers with GDM lack reliable sources of information, considered the disease as a minor and transient illness during pregnancy, and failed to realize the long-term risk of GDM and the protective effect of breastfeeding to themselves and their babies. They rarely considered GDM in their feeding decision. Instead, the formation of feeding behaviors depends on the balance between the benefits and barriers of breastfeeding as well as the level of social support.

**Conclusion:**

To promote breastfeeding, a multi-facet intervention targeted on healthcare providers (HCPs), GDM mothers and their networks was important to help GDM mothers better and correctly understand the disease and breastfeeding, and increase their capacity of breastfeeding.

## Introduction

Diabetes mellitus is a chronic and noninfectious condition, threatening global health and social economic worldwide. It is estimated that 7.2% of adults (aged 20-79) worldwide suffered from diabetes, resulting in 6.7 million deaths (roughly 12% of total deaths) in 2021 (1). Due to the huge burden and continuously rapid increase of diabetes among populations, diabetes has become one of the prioritized public health issues by World Health Organization (WHO) and countries globally (2).

As one important form of diabetes, gestational diabetes mellitus (GDM) is a common condition that women develop hyperglycemia during pregnancy. According to the International Diabetes Federation (IDF), it is estimated that roughly one-seventh pregnancy suffered from GDM worldwide, relating to 18 million births annually.

Although GDM often resolves after birth-giving, it has long-lasting health consequences to GDM mothers and their offspring (3). Studies have well established that GDM mothers are more likely to suffer from type II diabetes (T2DM) and cardiovascular disease (CVD) in their later life, with over half of them developing T2DM after delivery (4). Besides, the offspring of GDM mothers have a significant increased risk of obesity, CVD, T2DM and GDM in the future, which will form a vicious intergenerational cycle of diabetes and result in “infectious” health burden of the whole population.

Currently, lifestyle intervention is a widely-accepted treatment for GDM. Among them, early and exclusively breastfeeding (EBF) plays an important role. Studies have confirmed that exclusively breastfeeding can reduce the risks of obesity (5) and T2DM (6) in the offspring of GDM mothers It also prevents GDM patients from developing T2DM. Due to its benefits for both GDM patients and their infants, EBF has been highly encouraged in several guidelines (7) of the U.K, the U.S.A, China and etc.

However, the prevalence of breastfeeding among GDM mothers is far less than optimal. According to the WHO, only over one-third (34.8%) GDM mothers feed their infants with breastfeeding for 6 months after delivery, and the proportion is less in developing countries (8). Although few studies tried to improve GDM mothers’ breastfeeding rate and duration through education by healthcare providers (9) or peers (10), the effect is quite limited.

Existing studies have shown that mothers’ feeding practice is complex and many factors may sway their behavior, such as breastfeeding knowledge (11), attitudes (12), self-efficacy (13), perceived barriers of breastfeeding, personal characteristics (14), level of support from others (15) and etc. This situation is more complex for GDM mothers. Mothers with GDM are more likely to experience biological barriers for breastfeeding, such as delayed onset of lactogenesis II and cesarean section delivery, which was found associated with reduced adoption and duration of breastfeeding (16). Furthermore, mothers with GDM require more cognitive and social support of EBF due to physiological barriers (17), while studies have found that GDM mothers have inadequate support from healthcare providers and others, and reported less self-efficacy of breastfeeding compared with those without GDM (18). To address this issue, a comprehensive understanding of GDM mothers’ feeding behavior and its underlying determinants is important, which is still unclear with limited studies.

Thus, this study adopted a qualitative design, collected the experience of GDM patients from the beginning (disease diagnosis) to the end (forming stable feeding behavior), and aimed to explore how GDM mothers formed their feeding pattern based on Health Belief Model (HBM).

## Methods

### Study design

This study is a qualitative study based on semi-structured in-depth interviews with postnatal mothers who had been diagnosed with GDM during pregnancy in Wuhan, China. An interview approach was chosen due to its ability to deeply understand participants’ situation, experiences and beliefs, and to gain insights into the underlying determinants of their behaviors (19).

### Settings

The study was conducted in obstetrical department of Tongji Hospital, Tongji Medical College, Huazhong University of Science and Technology (Wuhan, China). Tongji hospital is one of the largest tertiary hospitals in central China and serves patients with acute and critical illnesses from all over Hubei Province and surrounding provinces. As the maternal critical care center designated by the government, the obstetrical department of Tongji Hospital has a total of 6 wards with over 200 beds. In 2021, Tongji Hospital serves approximately 6,800 pregnant women’s delivery, of whom 12% are diagnosed with GDM.

### Participants

China has established a nation-wide comprehensive prenatal care program, which is covered by maternity insurance for all pregnant women. Pregnant women are suggested to screen for potential GDM at the prenatal visit between 24 to 28 weeks based on oral glucose tolerance test (OGTT). The diagnostic criteria for fasting, one-hour and two-hour blood glucose (BG) is 5.1 mmol/L, 10.0 mmol/L, 8.5 mmol/L, respectively. Any result higher than the criteria is diagnosed as GDM.

To be included in the current study, participants were required to 1) be diagnosed with GDM and give birth no more than 6 months ago in Tongji Hospital; 2) did not have any type of diabetes or abnormal glucose tolerance before pregnancy; and 3) had no history of severe chronic diseases (cardiovascular, kidney and etc.), no severe postnatal complication, or contraindications to breastfeeding. Potential participants who were diagnosed with GDM and gave birth in Tongji Hospital during June to October, 2021 were reviewed. To maximize representation of GDM mothers with different characteristics, a purposive sampling was used, considering participants’ age, education, living places (urban/rural), occupation, pregnancy history, family structure (families living with mothers) and newborn’s characteristics (gender and age).

### Interview guideline

A semi-structured interview guideline was developed based on HBM with open-ended questions that focused on participants’ experiences and beliefs of GDM and how they developed current feeding behavior (20). Generally, HBM illustrates that whether an individual adopts a recommended health behavior depends on one’s belief in the effectiveness of the behavior together with one’s desire to avoid a potential illness (20). Probing questions were used when necessary for participants to elaborate their detailed experience and feelings regarding breastfeeding, which was not included based on HBM.

**Figure 1:**
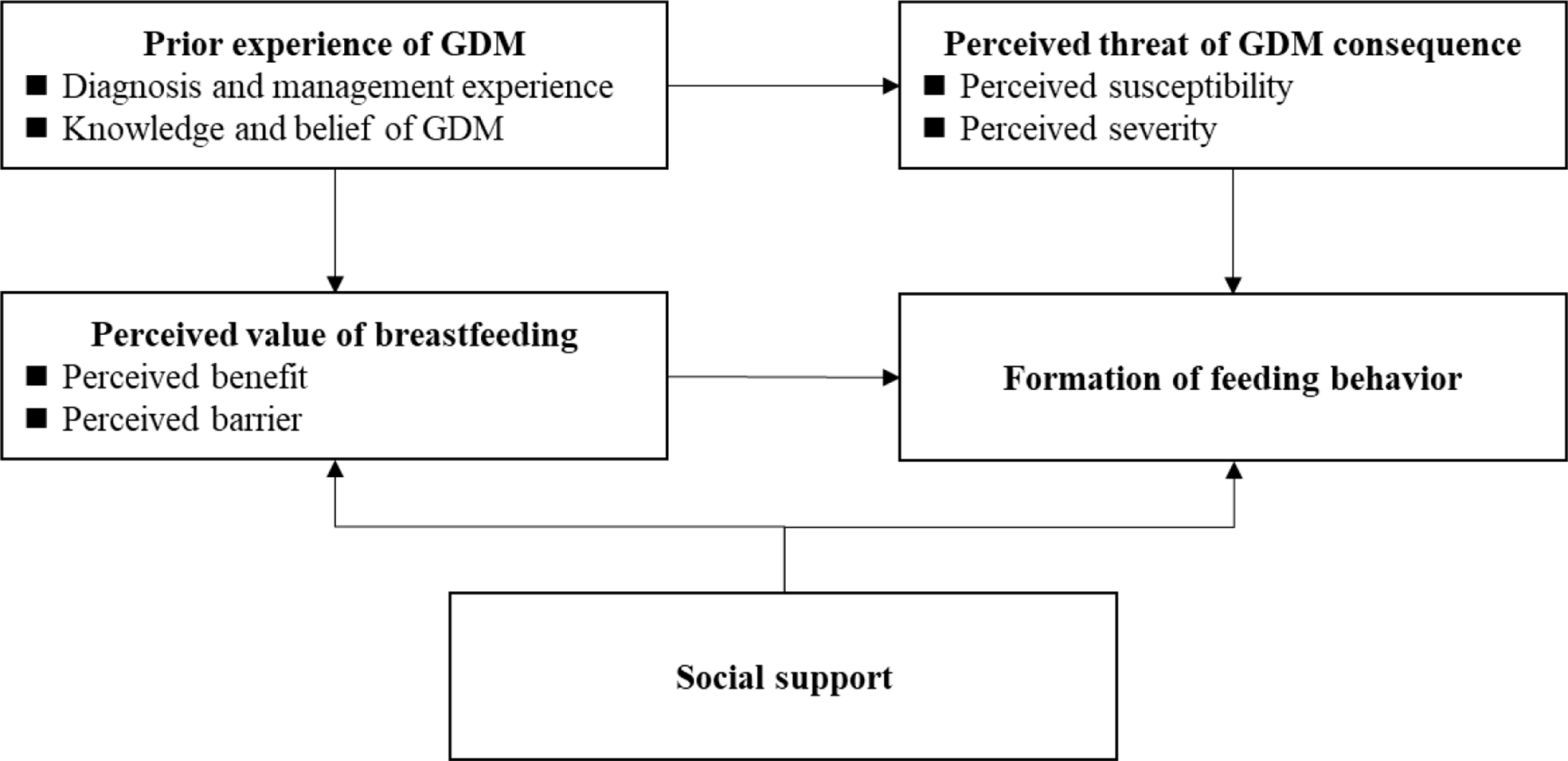
Framework of interview guideline based on HBM.

According to HBM (**Figure 1**), whether GDM mothers adopt breastfeeding mainly depends on their perceived value of breastfeeding and perceived threat of potential GDM consequences. Among them, the perceived value is a systematic evaluation of breastfeeding’s benefits and barriers, and the perceived threat is an overall assessment of susceptibility and severity of potential GDM consequence, respectively. In addition, the previous experience of GDM shapes their perceived threat of GDM consequence and perceived value of breastfeeding. Social support also influences feeding behaviors by changing mothers’ barriers of breastfeeding or directly changing mothers’ breastfeeding behaviors. Questions exploring the above topics were developed and used (**Box 1**). In the current study, future type II diabetes was chosen as GDM consequence due to its high prevalence globally and raising public awareness. Finally, participants’ demographics were also collected, including participants’ age, occupation, education, living place (urban/rural), way of delivery, smoking history, newborns’ age and gender. The interview guideline was qualified by several obstetric and breastfeeding professionals, who have regular contact with GDM patients for over 10 years.

Box 1 **Interview questions about GDM mothers based on HBM.**

Topic one: Experience of GDM
■ What are your experiences of GDM diagnosis and management during pregnancy?
■ What do you know about GDM?
Topic Two: Perceived threat of GDM consequence
■ Do you think you (or your child) may have type II diabetes in the future and why?
■ If you develop diabetes in the future, will you concern its consequence to you and your children?
Topic Three: Perceived value of breastfeeding
■ What do you know about the benefit of breastfeeding?
■ According to your experience, is there any barrier of breastfeeding?
Topic Four: Formation of feeding behaviors
■ How do you feed your child now and what do you consider when choosing this kind of feeding pattern?
■ What are your opinions about the support your received from others during your feeding?

### Data collection

Potential participants were screened by nurses at the obstetrical department of Tongji Hospital, and were informed about the study and invited to participate during the postnatal care follow-up. The interviews were performed by a pair of authors (PQ and LD) during November to December 2021 and each interview lasted for 22 to 43 minutes. Interviewers were postgraduate students in social medicine and health management and have the basic knowledge and skills of qualitative research and data collection. In addition, a 3-day training of the current study was conducted and all interviewers had passed a simulated interview test. Interviews were conducted face to face or by online video call according to participants’ preferences. All interviews were recorded with a digital audio recorder and then transcribed verbatim in Chinese in the same day. After 13 interviews, data saturation was met as estimated. Additional three interviews confirmed data saturation with no new information emerged. Finally, a total of 16 interviews were completed, including ten face-to-face interviews and six online video interviews, respectively.

### Data analysis

Theory-driven thematic analysis based on HBM was applied for data analysis, which included four main steps: data familiarization, initial coding, themes identification and themes reviewing. All transcribed text was first repeatedly read by researchers and as much as meaning units (a sequence of text) were initially coded during this stage. Multiple coding was encouraged and the same sequence of text was able to be classified into different codes to gain an overall and in-depth understanding of the data. Furthermore, a largely inductive approach was applied to classify the initial codes into several main themes based on HBM (see **Figure 1**). Emerged sub-themes were data-driven based on classification of initial codes within the same main themes. Finally, all identified themes and sub-themes were reviewed against the initial coding and transcribed texts to ensure that the whole text data was well represented and the relationships between themes were not distorted.

Two researchers (PQ and CL) independently conducted the thematic analysis. After each step, the results of two researchers were crosschecked and reconciled through negotiations. Disagreement was solved through discussions within the research team. To ensure credibility, results were sent to three participants for feedbacks and they confirmed the results were consistent with their experience of GDM and breastfeeding.

### Ethics

This study was supported by the National Natural Science Foundation of China (No. 71974061). The funding bodies were not involved in the design of the study, data collection, analyses, interpretation of data, or the writing of the manuscript. Written consent was obtained from each participant before the survey. We got permission for this study from Tongji hospital ethics review committee. The ethics approval number is NO.TJ-IRB20210755.

## Results

A total of sixteen women who had been diagnosed with GDM participated in the study. Their mean age was 31 years. For most women (81.25%), it was their first childbirth and half of all participants (n=8) gave birth by cesarean section. At the time of the interview, the average age of babies was 3 months and 10 days (100.3 days). More than half of mothers (n=9) used breastfeeding, some (n=6) used mixed feeding, and only one mother used artificial feeding, respectively. The demographic data of the participants is presented in **Table 1**.

**Table 1:** Demographic data of the participants.

	Age	Education	Type of delivery	Parity	Month of baby	Occupation	Members of family
1	29	Bachelor	Normal delivery	Firstborn	One	Secretary	Husband, parents in law
2	30	Associate	Normal delivery	Firstborn	One	Civil servant	Husband, babysitter
3	40	Associate	Cesarean	Second born	Two	Clerk	Husband, parents
4	29	Associate	Normal delivery	Firstborn	One	None	Husband, mother in law
5	34	Associate	Cesarean	Firstborn	Two	None	Mother in law
6	27	Associate	Normal delivery	Second born	Five	None	Babysitter
7	26	Associate	Cesarean	Firstborn	Five	Clerk	Husband, parents in law
8	29	Bachelor	Normal delivery	Firstborn	Six	Teacher	Husband
9	28	Associate	Normal delivery	Firstborn	Six	Clerk	Husband, parents in law
10	40	Bachelor	Cesarean	Firstborn	Six	Teacher	Parents
11	32	Bachelor	Normal delivery	Firstborn	Two	Financial staff	Parents
12	28	Associate	Normal delivery	Firstborn	Two	Secretary	Husband, mother in law
13	31	graduate	Cesarean	Firstborn	Four	Teacher	Husband
14	39	Middle school	Cesarean	Second born	Two	None	Husband
15	28	Vocational school	Cesarean	Firstborn	Five	None	Husband, mother in law
16	26	Master	Cesarean	Firstborn	Two	Teacher	Husband, babysitter

### GDM diagnosis and control during pregnancy

*GDM diagnosis and perceived severity*

*Question:”what are your experiences of GDM diagnosis and management during pregnancy?”*

Expect for one participant who experienced dizziness and sought medical care, no one else had perceived any symptom associated with GDM and the condition was diagnosed during pre-natal oral glucose tolerance test (OGTT). When informed of the results, most women attributed it to the occasional high-carbohydrate food before the test (such as cake and beverages) or unhealthy eating habits during the pregnancy (such as overeating and regular high-sugar fruit intake).

*I was not surprised (about the result). I didn’t understand the examination very well. On the day before the test, I ate a large piece of cake at 10:00 that night and I had told doctors about this. (No. 13)*

However, for those who believed they had adopted healthy eating behaviors, confusion remained and they simply attributed GDM to the pregnancy.

*I don’t know why. I’m not fat and nobody in my family is fat. There is no medical history of GDM in my family. My family was also surprised at why I had this. It may be the effect of hormones in the body due to pregnancy. (No. 1)*

Irrespective of disease attribution, participants assumed GDM as a transient disease, which would either be ended by diet control or on its own after the childbirth.

Initially, all mothers thought GDM was not serious based on the numbers of abnormal indicators (only one of the three parameters were abnormal) and to what extent anomalies deviated from reference values (the value was a little higher than normal range).

*I felt that only one fasting blood sugar was high. The latter two were normal and it should not be a big problem. (No. 16)*

GDM mothers were only concerned that the disease would affect their diets. They also cited the examples of other women with GDM around them or on the Internet to indicate that GDM were prevalent. Participants also compared their conditions with those with extremely high blood glucose, justifying that their illness was not serious.

Such a view of GDM was deeply ingrained. When doctors tried to explain that GDM, even with a slightly raised blood glucose, was also serious, participants interpreted this as an exaggeration on the doctors’ behalf for patients to pay attention to the disease.

*I thought doctors often said something a little more serious, because if he doesn’t say like this, you can’t take it seriously at all. I told him it’s just a little bit high. But he said that even if it was 0.1 or 0.01 higher, the figure was abnormal. He also said that if I cannot control it, it will continue to rise. (No. 4)*

Mothers also relied on their healthcare providers’ behavior to judge the severity of the disease. Most doctors simply advised participants to control their blood glucose, and mothers perceived the limited emphasis on the disease, which meant that GDM was not serious.

*I was referred to the specialized gestational diabetes unit. However, it is a very simple and small office, which people often mistake as the doorman. After entering, the doctor didn’t say much to me. He told me how much to eat every day, eat eggs and corn in the morning, and monitor blood sugar. It seems that the hospital didn’t pay much attention to the disease and it is funny to set a small office for this (disease). (No.16)*

However, the assessment of the severity of GDM is dynamic and continuing throughout the entire pregnancy. Participants mentioned blood glucose monitoring and fetal characteristics as indicators to assess the severity of GDM. GDM was considered severe, when the numbers of abnormal indicators exceed one, one’s perception that blood glucose was much higher than normal range, or if abnormal results of OGTT persisted or worsened after diet control.

*At first, I was not serious about the disease, and just listened to the doctor to control blood sugar. Then I found that it was not under control after a month. You know, I was a little sensitive during pregnancy and became more anxious since the blood sugar stayed high. I also worried that my baby would be deficient in nutrition. (No.8)*

On the other hand, the occurrence of specific fetal conditions, including macrosomia and polyhydramnios, significantly contributed to the assessment of severity of GDM since they implied severe adverse events, such as fetal malformation.

### Information searching and knowledge regarding GDM

Since GDM was regarded as “not serious”, participants rarely collected information and displayed limited knowledge of GDM. The main concerns were about how to control their blood glucose as well as the outcomes of uncontrolled GDM. Therefore, participants mainly searched for ways to change their diets and the outcomes of uncontrolled GDM on fetus. The frequently mentioned consequences of uncontrolled GDM were oversized fetuses or being overweight. Other adverse effects included neonatal high/low blood glucose, fetal developmental retardation, polyhydramnios and premature delivery.

Regarding the sources of the knowledge, healthcare providers and the testimony of other pregnant women with GDM were their main sources. However, healthcare providers played a limited role. For most doctors, they merely informed participants about their results of OGTT and reminded participants to monitor and control their blood sugar, for example, eat coarse grains and control the intake of high-sugar food.

*-Have the doctors or nurses told you anything about GDM?*

*-No, they didn’t tell me anything. They just look at the results and tell you whether it was normal or not. They told me I’m OK and I did not talk much with them. Only in the later stages of pregnancy, I went to a tertiary hospital to see a professor.*

*-Has the professor told you anything?*

*-No. He just said my blood sugar was a little higher and I should control my diet. We did not talk much about this. (No.16)*

However, without interpretation, participants were confused about what to eat. Some doctors also tried to educate participants about the consequences of uncontrolled GDM. However, insufficient interpretation also hindered their understanding.

For experience of other GDM women, community-based websites and mobile applications were the main sources of information, from which participants learnt to manage blood glucose. No participants questioned the information since they thought the sharers had similar experience with them. Such community not only educated participants on how to manage GDM, but also provided psychological comforts. However, the severe adverse experiences shared in these communities also lead to increased maternal anxiety.

*You know that Tik-Tok ‘kills’ people, right? I saw that a pregnant woman who ate two kilograms of litchis and her blood sugar was too high, resulting in a stillborn. I had anxiety for days. (No.4).*

### Management of GDM

In terms of GDM management, the first target of all participants was diet control. They had two main approaches, namely, changing dietary structures (replace rice/flour with coarse grains as staple food) and reducing total intakes. In addition, perceived high-sugar food were also strictly controlled, including beverages, desserts and fruits. One participant also mentioned a change in cooking style. However, the attitudes towards diet control were polarized. Some of participants admitted having increased stress due to diet control, while the rest appreciated that such change helped them adopt a healthy lifestyle.

Eleven participants also conducted blood glucose monitoring. However, most of them only monitored the abnormal value according to their anomalous OGTT results (fasting, one-hour or two-hour) and the frequency was arbitrary. Participants used the monitoring results to continuously assess the severity of GDM and effectiveness of the adopted management.

For a few of participants (n=3), the monitoring was brief, since they perceived limited value of this behavior (blood glucose stabilized at the normal range after a period of diet control) or they perceived the process as painful. One woman also misinterpreted “fasting”, thinking that drinking water is forbidden before monitoring, and thus abandoned this behavior.

*I did monitor (blood glucose) for a while, and then I felt that my fasting blood sugar seemed to stabilize around the upper limit of the normal range and I felt no symptoms. Thus, I continued to eat as usual, and gave up monitoring (No.7).*

*I didn’t check one- and two-hour blood sugar. I only checked fasting glucose every day. I’m afraid of testing and my husband helps me. However, he has to work. So, I just take fasting blood sugar every day. If the result is sometimes slightly higher, I know that I cannot eat a sweet fruit. Then, after a week, it will come back to normal. (No.4)*

Half of participants also mentioned 30-60 minutes of walking after meal as regular exercise. On the contrary, the rest stated that they did not adopt this method due to restrictions such as time, fetal characteristics (placenta previa) or personal unwillingness. Drugs were rarely proposed by the participants (n=2).

The management of GDM was also dynamic and reflected on the perceived GDM severity. Reduced severity relaxed participants’ diet control, induced monitoring cessation, and sometimes led to non-adherence to medical treatment. Otherwise, as severity increased, management of GDM were more strictly followed. Five participants also reduced diet control in the last month of pregnancy, for which they justified as potential fear of fetal malnutrition and closing preparation for birth giving.

*The last month before giving birth, I didn’t have the injection of insulin. At that time, I monitored my blood sugar and it stabilized at roughly 5, no matter if I took my injection or not. Thus, I did not take insulin, because it’s okay in the last month that fasting blood sugar stables at 5 to 7 after meal. At that time, I thought the blood sugar was good after diet control and the dose (of insulin) was quite small, just three units (No.1).*

*In the last month, I started to eat what I want. It doesn’t mean that I’m going to eat sugar, just vegetables and rice, to make my fetus grow faster and well (No.9).*

### Perceived threat of future diabetes

*Question: “Do you think you (or your child) may have type Ⅱ diabetes in the future and why?”*

#### Perceived susceptibility

When asked about the susceptibility of future diabetes, over half of participants (N=10) thought they had higher risk of developing diabetes in the future compared with general populations. Participants talked about family history of diabetes, personal obesity and lifestyle to estimate the likelihood of future diabetes. These principles were applied equally to their babies, and it was believed that the risk was tiny, since they thought the newborns would be brought up in a healthier lifestyle.

*According to the conditions of my family, I think there is at least 50% possibility for me to get diabetes. There is a history of diabetes in my mother’s family. My mother, my uncle and some of my cousins all have diabetes. (No.3)*

Most participants (n=10) were not aware of the relationship between GDM and future possibility of diabetes. It was widely believed that GDM was a transient condition due to pregnancy or temporary unhealthy diet during pregnancy, and it had no impact on themselves and their babies’ future. This belief was so prevalent that most participants did not undergo medical review after giving birth (n=13). A few of participants reviewed their blood glucose and further strengthened their belief that GDM was transient since their blood glucose had normalized (n=3).

*Diabetes, in my opinion, is a disease that patients have. I’m different. GDM is caused by pregnancy. It is not diabetes, because it will become normal after giving birth (No.14)*

*I checked my blood sugar after giving birth and it became normal. I don’t have any symptoms now, and I will continue to control it. I am afraid of developing diabetes.… I have read on the Internet that there are two possibilities of diabetes. One is hereditary, and the other is hormonal change due to pregnancy. I have returned to normal now, so I think my diabetes won’t pass to my child. (No.5)*

Six participants had heard that GDM increased their risk of future diabetes. However, they assumed that since their GDM was not severe, there was no need to worry about its consequences.

#### Perceived severity

*Question: “If you develop diabetes in the future, will you concern its consequence to you and your children?”*

In terms of severity of future diabetes for participants and their newborns, personal assessment of diabetes’ impact on diet and whether there was serious complication were two main criteria. Participants compared other diabetic patients’ experience to their own in terms of diet control during gestation to obtain an estimation of severity.

It is generally believed that diabetes was a severe disease, and if uncontrolled, diabetics were at risk of severe complications or required insulin injection. On the other hand, patients who succeed in maintaining blood glucose control do not have serious conditions, resulting in less severe assessment of diabetes.

Likewise, the attitude towards diet was another important indicator. Women who thought eating is an important part of life and perceived diet control as hard, resulted in a higher severity of diabetes, and vice versa. In addition, for those who perceived diet control as not hard, the experience of diet control during pregnancy increased their confidence to cope with future diabetes and they appreciated that GDM raised their awareness on unhealthy eating.

*It (diabetes) definitely influenced my live. There are many things that you can’t eat, and you can’t live and eat normally, right? First and foremost, the thing that breaks me the most is diet at that time (during pregnancy). The most annoying thing is that you barely eat and there are still a lot of things you can’t eat. At that time (during pregnancy), I just ate a little bit and I was very hungry. I only ate vegetables every day and I could not eat salt, oil and so much more …. (No.10)*

*After GDM, I found that my weight would not increase a lot with strict diet control. It is quite healthy for my body. Yesterday I told myself that I will strictly control my diet in the future. And I should be healthy and not obese (No.5).*

However, no matter whether diabetes was assessed as serious or not, participants generally did not worry about future diagnosis of diabetes. They argued that it was too early to worry about this issue. In addition, mothers were generally optimistic that they were able to succeed on maintaining healthy lifestyle to reduce the probability of future diabetes and the occurrence of serious complications. As for their children, healthy living style would be cultivated as soon as possible to prevent the emergence of diabetes at an early age. Even if they or their children had diabetes, it would not be serious as long as it was well controlled.

*Diabetes is actually not a terrible disease. But you need to eat less food. The main things are to eat less and exercise more. It will be fine if you eat less and exercise more. (No.3)*

### Perceived benefits and barriers of breastfeeding

#### Perceived benefits

*Question: “What do you know about the benefit of breastfeeding?”*

The participants widely accepted that breastfeeding was very good for their children. However, that understanding was superficial. The commonly perceived benefits of breastfeeding were rich in nutrition, able to strengthen newborn’s immunity, safety without additives, and cost-saving. In addition, three mothers also mentioned the benefits of breastfeeding for themselves, for example, helping postpartum recovery and improving the mother-child bond.

Almost all participants had not heard of the benefit of GDM mothers’ breastfeeding on preventing both themselves and their babies from future diabetes for (n=15). Though one participant was aware of the relationship, she believed that the breast milk of GDM mothers was unhealthy for the newborns since it had higher sugar content.

*Before I breastfeed, I have checked my milk. It seemed that the sugar of my milk is a bit high. And I’m worried about the possibility that my child will have low blood sugar in the future. It seemed that a long-term intake of high-sugar (food) causes a large amount of insulin secretion that can cause his low blood sugar. (No.11)*

When informed that breastfeeding in GDM was able to reduce the risk of future diabetes for themselves and their babies, most participants (n=10) doubted it and affirmed that there was no relationship between feeding patterns and diabetes.

*I don’t think so. I don’t know. Does breastfeeding really have such benefit? (No.5)*

Among mothers who doubted information’s credibility, two participants inferred that such information would not change their feeding patterns even if the information was true. The main concern was the huge costs of time and effort of breastfeeding. One indicated that if breastfeeding was able to exclusively reduce their newborns’ likelihood of diabetes, she might try to change her feeding behavior and extend the duration of breastfeeding.

For a few mothers (n=6) who believed in the information that GDM mothers’ breastfeeding was able to reduce the risk of future diabetes of themselves and their babies, such information strengthened their intention to perform breastfeeding (n=3), extend the duration of breastfeeding (n=2), and increase the volume of breast milk (n=2).

*-Would such information (breastfeeding reduce mothers’ and newborns’ likelihood of future diabetes) change your feeding behaviors?*

*-Yes. I’ll be more decidable in breastfeeding. Before birth giving, I had decided to breastfeed if I have a good condition of breast. I think that I would breastfeed my son one year, at least one year, I think I will.*

#### Perceived barriers

*Question: “According to your experience, is there any barrier of breastfeeding?”*

The main barrier of breastfeeding was insufficient breast milk. Mothers mentioned that the newborns kept drinking and crying, which they perceived as an inability to keep up with feeding demand.

Concerning how to increase the amount of breast milk, participants commonly turned to traditional remedies and statements from other mothers, social networks and the Internet. Drinking various kinds of concoctions were commonly mentioned. However, these methods were not always effective. Lactation experts were also referred to some participants and only one mother mentioned that frequent baby’s sucking was able to increase the amount of breast milk.

*After I come home, my mother often cooks foods that stimulate milk, such as soup of crucian carp, pig trotters, vegetables, etc.… I used to, (after birth giving), drink Chinese herbal tea and produced less breast milk. (No.6)*

Another barrier was the huge burden of breastfeeding. Participants admitted that breastfeeding was time-consuming and there were short intervals between them (high frequency), costing mothers’ consequent time and energy. This situation was more prominent at night. Mothers complained that they had to get up and feed frequently, resulting in a lack of sleep for both mothers and their newborns. Participants believed that the lack of sleep led to insufficient milk production, and the lack of sleep in newborns resulted in growth retardation. In contrast, feeding with formula led to newborn’s longer sleep and gave mothers time to rest. Thus, those who found themselves unable to manage the burden of breastfeeding, preferred artificial feeding instead.

*I can’t stand breastfeeding all day. I tried once. She (the baby) eats for a while and sleep for a while. It’s too frequent. I feel that both of us are tired. Later, I found that feeding with formula made her sleep a little longer and I was afraid that she wasn’t getting enough sleep before. People always say that children grow up during sleep. (No.1)*

Whether mothers had to work was an important factor influencing participants’ self-assessment whether they were able to cope with breastfeeding. Since working itself required time, breastfeeding could not be easily achieved due to limited supporting facility in working environments, for example, lactation room. In addition, breast milk expression during work was more time- and effort-costing. Along with the reduced frequency of breastfeeding, once mothers returned to work, the amount of breast milk decreased and mothers had to change their feeding patterns. For those who were able to nurse their babies full time, breastfeeding was easier.

*I initially planned to breastfeed for 6 months, but I have to spend at least an hour and a half to go to work. Thus, I cannot come home at noon. I have to work outside all the day. … Thus, I might slowly reduce breastfeeding after I go to work, and then (feed by) formula (No.12)*

In addition, misunderstandings that breastfeeding caused neonatal jaundice also induced a change in feeding patterns, which mothers called “breast-feeding jaundice”. They stated that doctors recommended them to suspend breastfeeding to observe whether there was a decrease of bilirubin level, which was interpreted as breastfeeding contributing to the high bilirubin level. They also stated that they had to reduce the bilirubin level in a short time to enroll in the national neonatal immunization program.

*When we got diagnosed with jaundice, the doctors said that we should not breastfeed for a while and observe whether the jaundice comes down. (No.2)*

*Because my baby had jaundice in the first three months, I used mixed feeding, because jaundice was caused by breast milk, so I insisted on mixed feeding. (No.7)*

*The jaundice was found on physical examination. It (bilirubin level) was over eight. He needed lower levels to have vaccination, right? He was born prematurely and he did not have vaccination before (No.2)*

Another misunderstanding reported by a few of mothers(n=2) was that breast milk was nonnutritive after six months of feeding, which induced early cessation of breastfeeding. Even though a few of mothers(n=3) mentioned that WHO recommends breastfeeding for two years, mothers rarely adopted it. Mothers mentioned that lengthy breastfeeding deprived child’s opportunity to learn other things. They believed that boys could be “gender-conscious” with too long breastfeeding.

Other barriers referred by participants included breast conditions (pain, blocked ducts, breast distension, mastitis), baby’s refusal of breast milk, belief that formula milk was as good as breast milk and mother’s concern about body shape.

### Forming feeding behaviors and social support

#### Decision making process of how to feed

*Question: “How do you feed your child now and what do you consider when choosing this kind of feeding pattern?”*

All mothers stated that they had specific feeding expectation before giving birth, with twelve mothers planning to feed by breast, three intending mixed feeding and one mother expecting formula, respectively.

Generally, mothers had not intentionally gathered knowledge regarding feeding. Their expectations were mainly shaped by surroundings, including family, friends, healthcare providers, confinement attendants and the Internet. Participants relied on experiences from family, friends and mothers’ community (online or offline) to choose the type of feeding. They perceived a strong incentive for breastfeeding from “lactation specialists”, including healthcare providers (doctors and nurses), breast massagers and confinement maids, which shaped their feeding decision.

*Before feeding, other people and my family all said that breast milk is nutritious and good for baby’s immunity. Basically, I had been pressured into breastfeeding, you know? I think breastfeeding is good for me and children. Thus, as long as there is milk, I will breastfeed my baby. Before giving birth to him, I had already decided for exclusive breastfeeding. (No.4)*

In the early days after childbirth, feeding behavior fluctuated along with the dynamics between the mother and newborns, and gradually formed a relatively stable feeding pattern. During the process, the adoption of breastfeeding was based on a balance where both the newborns and the mothers could achieve maximum benefit. Mothers generally believed breastfeeding was better than formulas, and tried to keep up with needs of newborns as much as possible (refer to the above perceived benefits). Mothers had to bear the burden caused by breastfeeding and assessed whether they would be able to manage it (see above perceived barriers). Once a mother perceived the value of breastfeeding and self-assessed that she was able to manage the burden, a stable breastfeeding behavior was achieved.

*-What is the main barrier of breastfeeding?*

*-Not enough breast milk.*

*-If you can increase the amount of breast milk by more frequent breastfeeding all the day, would you like to do this?*

*-No. I think it is impossible for me to be with the baby all the time. I have to rest. Mixed feeding is more suitable for me. (No.7)*

#### Social support form feeding behaviors

*Question: “What are your opinions about the support your received from others during feeding?”*

Social supports also played an important role in the formation of feeding behavior. Healthcare providers rarely presented knowledge and skills regarding breastfeeding, and there was limited communication between doctors and patients. Mothers often could not understand how to feed and how to deal with early insufficient milk and breast congestion, which resulted in mixed feeding in the early stage.

For most participants (n=9), they sought help from confinement maids and lactation professionals. Mothers stated they learnt how to correctly feed based on practices of the latter. However, not all of them were qualified and there was some incorrect knowledge of breastfeeding identified.

*The confinement maid helped me a lot with feeding. I was relieved when she was around me. If my baby cries, she helps me to feed her, by putting my nipple into her mouth … Since I have a lot of breast milk, there was a blocked duct one time.… I was totally dependent on the confinement maid at that time and did not pay much attention to feeding. (No.5)*

*My mum thinks that breast milk is not nutritious after 6 months. One day, she asked the confinement maid and was informed that breast milk will not be nutritious after 6 months. My mum is more confident and suggests me to breastfeed till then.*

After the feeding behaviors become stable, family supports help mothers with food preparation and child care. Most mothers appreciated the help since it decreased their burden. However, a few of mothers(n=4) complained that overt concern of the newborn aroused their disgust. The worry of insufficient breast milk existed not only among mothers but also in the family. One participant indicated that family members may secretly feed the newborn by formulas if they perceived the newborn was not stuffed. In the end, regardless of the social support, mothers prevalently had the final decision of adopting feeding pattern.

*When my child was two months old, my mother-in-law fed my child by formulas secretly, not even water, she gave him milk powder! My child was young, she (mother-in-law) was afraid that the child was hungry and thought he should drink (formula milk) when hungry. (No.7)*

### Modified health belief model for GDM mother’s breastfeeding decision

**Figure 2:**
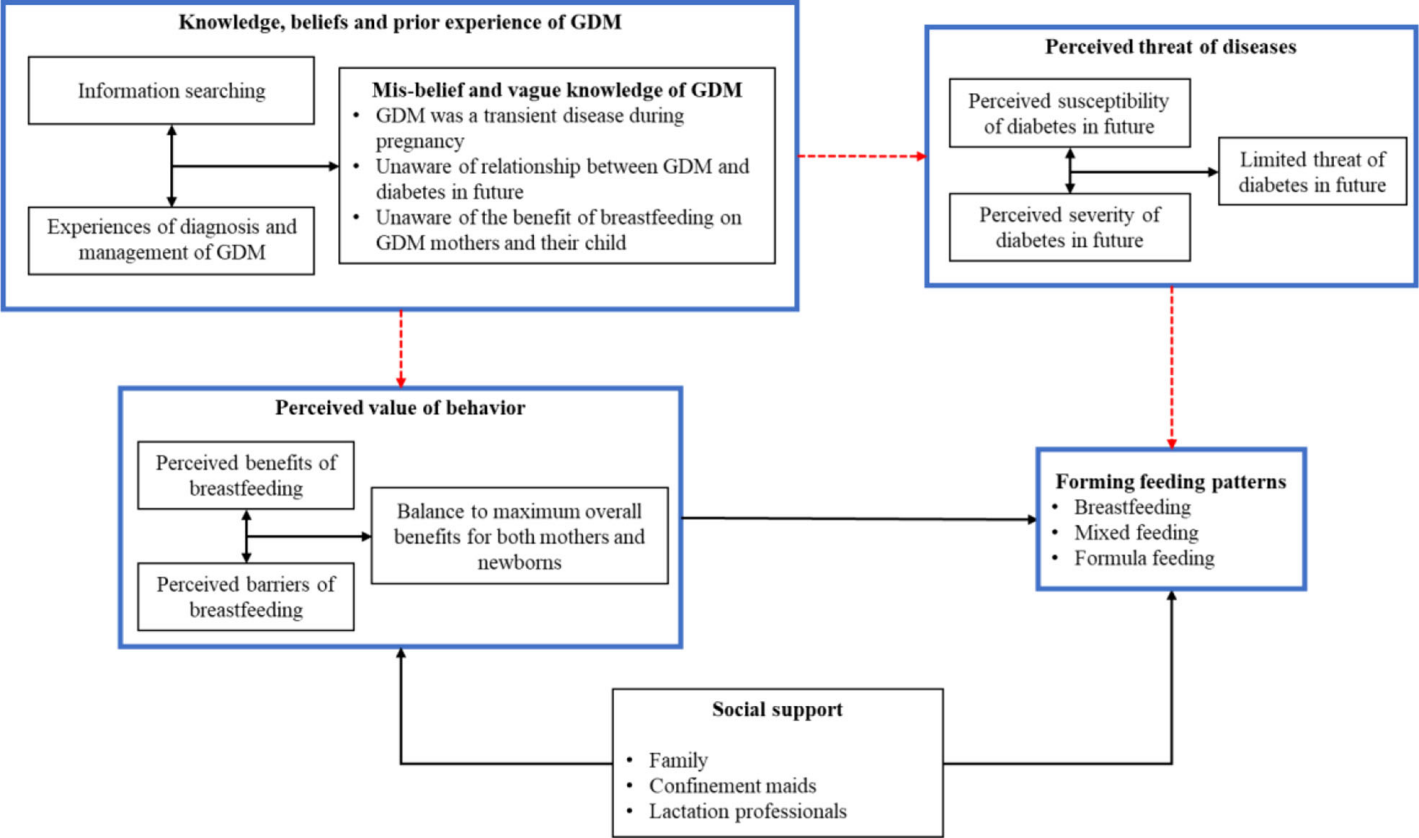
Decision-making process of GDM mothers’ feeding behaviors.

Derived from HBM, **Figure 2** presents the decision-making process of GDM mothers’ feeding behaviors. The model showed that mothers wrongly believed that GDM was a transient disease only limited to pregnancy, were unaware of its long-term effect for diabetes in the future and did not recognize the benefit of breastfeeding in preventing it. The misconceptions and vague knowledge deterred their cognition on their (and their child’s) susceptibility to diabetes and benefits of breastfeeding (the red dotted line in **Figure 2**). Thus, the experiences of GDM played a limited role in feeding decision. Instead, GDM mothers balanced the benefits and barriers of breastfeeding to maximize the overall benefits for both themselves and their child. In addition, social support also helped form feeding decision by releasing the barriers of breastfeeding. When a mother self-assessed that she will be able to manage the barriers and perceived the positive value of breastfeeding, stable breastfeeding behavior could be achieved.

## Discussion

### Main findings

This study adopted a qulitative design to explore how GDM mothers developed their feeding behaviors. Based on HBM, mothers’ experiences of GDM, their perceived threats of GDM consequence, perceived values of breastfeeding, received social supports of feeding and how these factors influence their feeding decision were together attended into forming an overall understanding of breastfeeding decision-making process.

Due to a lack of education and a reliable source for appropriate management of GDM, mothers with GDM had a mis-belief and vague knowledge of the disease, generally considering it as a minor, transient illness during pregnancy and holding sub-optimal understanding of its consequences and management. This situation hindered GDM mothers from realizing the potential risk of the long-term health burden of GDM and potential protective effects of breastfeeding to themselves and their babies.

In the current study, GDM mothers rarely considered GDM in their feeding decision. Instead, whether mothers adopted breastfeeding depends on her evaluation between the benefit and barriers (burden) of breastfeeding for both herself and the newborn. Social supports from family and other professionals help GDM mothers perceive the benefit or reduce the barrier, which resulted in promotion of breastfeeding.

### Strength and limitations

To our best knowledge, this was the first study based on a qulitative design to explore how GDM mothers developed their feeding behaviors in developing countries. The adoption of HBM model enables us to comprehensively understand potential determinants and how these factors influence GDM mothers’ decision-making of breastfeeding. Existing studies often referred healthy mothers’ decision-making process of breastfeeding and generalized it into GDM mothers, which was inconsistent with GDM mothers’ experience and ignored some determinants of behaviors, such as prior experience contacting with healthcare system and higher level perceiption of future threat and etc.

However, this study is not free of limitations.All GDM patients were recruited in one tertiary hospital in middle area of China and the generalization of the current result to other areas should be careful. Though we adopted a purposive sampling to cover as various background of GDM patients as possible, the mothers with formula feeding seemed underrepresentive in the current study.

### Comparison to existing studies

Unlike other studies showing that GDM increased women’s anxiety and stress, and patients struggled to cope with the disease (21), most participants in the current study considered that GDM was not a severe illness and their management was arbitrary. Early monitoring abandon, unstrict diet control and even non-indication change of drug use were identified, which was associated with severe adverse events of GDM patients and their fetus, such as pre-eclampsia, amniotic fluid pollution and premature rupture of membranes (22). GDM patients formed their own beliefs and behaviors based on the contacts with health professionals (23) and other patients (21). In the current study, health professionals’ neglectful attitude and practices shaped patients’ indifferent attitudes and practices towards GDM.

On the other hand, support from healthcare providers is particularly important for GDM women, as they are expected to experience more problems with breastfeeding due to physiological barriers (16). The current study showed that GDM patients were un-well, if not, supported by health professionals in China, consistent with the previous studies in other developing areas (21, 24). Though GDM screening was suggested and covered by the national prenatal care programme for pregnant women in China, the poor communication and prevalent neglect of health professionals hinders its effect. This was against the fact that Chinese guideline recommended several education and management strategies for GDM. Healthcare providers’ prevalent neglect of GDM, non-adherence to the guideliens and poor communication all played a role. In the case of GDM, these factors led to rare education with insufficient interpretation for GDM patients, resulting in sub-optimal knowledge and management of the disease, unawareness of GDM long-term threat and underestimation of the protective effect of breastfeeding for themselves and their offspring.

GDM patients’ sub-optimal understanding of the disease and breastfeeding were prevalent worldwide, which was also identified in Polish (25), Bangladesh (26) and India (27). Due to limited knowledge, mothers rarely considered GDM in their decision no matter what breastfeeding patterns they chose according to the current study. Previous studies had showed that increased awareness of breastfeeding’s benefits and more positive attitudes towards breastfeeding resulted in higher intention (28) and less discontinuing of breastfeeding (29), so it was plausible to further promote GDM mothers breastfeeding through improving their knowledge.

However, even with increased knowledge of GDM, there was still a knowledge-behaivor gap (30). The current study showed that GDM mothers are prevalently optimistic about their ability to cope with future diabetes of themselves or their offspring. This situation may reduce their risk perception and hinder their adoption of recommended behavior to avoid potential health threat. This situation was identified in GDM patients’ postnatal behaviors. Even with awareness of diabetic risk, studies have shown that GDM patients may normalized diabetes based on their family history of diabetes and reduced their fear of future disease, resulting in less health-attending behaviors and reviews after delivery (30, 31)

Consistent with existing studies, insufficient milk and potential burden were two main barriers for GDM mothers’ breastfeeding. A study from Australia showed that breastfeeding problems and mothers’ return to work was associated with early cessation of breastfeeding (≤3 months) (32). Among them, insufficient milk supply was the most common mentioned issue by GDM mothers (32). However, there is no significant difference between GDM mothers and healthy mothers in the prevalence of insufficient milk supply (33) in the U.S.A, and GDM mothers seemed more likely to report that their offspring had limited interest in breastfeeding (34). Future studies were warrented to investigate whether there is an increase of prevalence of insufficient milk supply in GDM mothers and whether this was associated with less adoption of breastfeeding.

Social support plays an important role in mothers’ decision-making of breastfeeding. According to the current study, it was possible to influence mothers’ feeding behaviors by reducing or increasing the barriers of breastfeeding. For GDM patients, most support promoted the adoption of breastfeeding, which was consistent with the result that adequent social support resulted in less early cessation of breastfeeding (32). However, unqualified social support sometimes exerted extra barriers, for example, mis-understanding that breast milk is not nutritious after six months and contradictory feeding behaviors between mothers and family members in the current study. Doughty and colleagues found similar result that GDM mothers reported their husbands’ preference for formula feeding (34), suggesting higher level of barriers towards social support for breastfeeding.

### Implication for clinical practices and policy

This study highlighted several factors to be targeted in the development of interventions, and suggested that a multi-facets intervention for Health care providers (HCPs), GDM mothers and their support network was more likely to promote GDM mothers’ breastfeeding.

Firstly, HCPs’ education is fundemental to ensure that GDM patients receive recommended management according to the clinical guideline and form a correct understanding of the disease. Raising awareness of GDM, improving disease management and training of communication skills are of great importance.

Secondly, education on GDM mothers about breastfeeding knowledge and skills was also of great significance. Studies from U.S.A (35) and China (35) illustrated that breastfeeding education *via* classes and text messages throughout prenatal and postnatal stages significantly increase GDM mothers’ likelihood to adopt breastfeeding and extend their breastfeeding duration. Due to the fact that HCPs served as the authority to form the public’s behaviors related to health, doctors, nurses and midwives all may play a role.

Finally, though social support was expected to promote GDM mothers’ breastfeeding, in the current study, there is unqualified and inappropriate support exerting extra barriers and hindering GDM mothers breastfeeding. Besides the general education towardes the public to bulid a breastfeeding promotion culture, targeted education regarding breastfeeding should be extended into GDM mothers network, including family members and maternity matron. However, future studies are warrented to confirm whether these interventions are able to promote GDM mothers breastfeeding.

## Conclusion

Based on HBM, this study comprehensively explored GDM mothers’ experience of the disease and how they develop feeding behaviors. GDM knowledge, perceived threat of GDM consequence, benefit and barriers of breastfeeding and social support were deeply attended into based on a qualitative design. Mothers with GDM generally considered the disease as a minor and transient illness during pregnancy, and thus failed to realize the long-term risk of GDM and protective effects of breastfeeding to themselves and their babies. They rarely considered GDM in their feeding decision. Instead, the formation of feeding behaviors depends on the balance between benefits and barriers of breastfeeding and the level of social support. Thus, multi-facets interventions on HCPs, GDM mothers and their networks were important to help GDM mothers form a correct understanding of the disease and breastfeeding, and increase their capacity of breastfeeding.

## Data availability statement

The raw data supporting the conclusions of this article will be made available by the authors, without undue reservation.

## Ethics statement

The studies involving human participants were reviewed and approved by Tongji Hospital, Tongji Medical College, Huazhong University of Science and Technology, Wuhan, Hubei, China. The patients/participants provided their written informed consent to participate in this study.

## Author contributions

PQ: performed the data collection,contributed to the writingg of the manuscript. CL:conceived and designed the study, contributed to the writing of the manuscript. TZ: conceived and designed the study. LD: performed the data collection and analysis. RL: performed the data collection. DW: conceived and designed the study. XD: performed the data analysis. All authors contributed to the article and approved the submitted version.

## Conflict of interest

The authors declare that the research was conducted in the absence of any commercial or financial relationships that could be construed as a potential conflict of interest.

## Publisher’s note

All claims expressed in this article are solely those of the authors and do not necessarily represent those of their affiliated organizations, or those of the publisher, the editors and the reviewers. Any product that may be evaluated in this article, or claim that may be made by its manufacturer, is not guaranteed or endorsed by the publisher.
